# Relationship between leisure-time physical activity and depressive symptoms under different levels of dietary inflammatory index

**DOI:** 10.3389/fnut.2022.983511

**Published:** 2022-09-07

**Authors:** Yanwei You, Yuquan Chen, Jiahui Yin, Zheng Zhang, Kening Zhang, Jing Zhou, Shuai Jin

**Affiliations:** ^1^Division of Sports Science and Physical Education, Tsinghua University, Beijing, China; ^2^School of Social Sciences, Tsinghua University, Beijing, China; ^3^Institute of Medical Information/Medical Library, Chinese Academy of Medical Sciences & Peking Union Medical College, Beijing, China; ^4^College of Traditional Chinese Medicine, Shandong University of Traditional Chinese Medicine, Jinan, China; ^5^School of Clinical Medicine, Tsinghua University, Beijing, China; ^6^Department of Nutrition and Food Hygiene, Public Health College, Harbin Medical University, Harbin, China; ^7^Catering Service Center, Tsinghua University, Beijing, China; ^8^College of Big Health, Guizhou Medical University, Guiyang, China

**Keywords:** leisure-time physical activity, depressive symptoms, dietary inflammatory index, cross-sectional study, mediation analyses

## Abstract

Depressive symptoms are major public health problems. Leisure-time Physical activity (LPA) and dietary inflammatory preference are emerging factors that tends to affect the mental health status. There is limited evidence regarding the joint influence of LPA and dietary status on the prevalence of depression. This study was a cross-sectional study, which used a nationwide represented sample from the National Health and Nutrition Examination Survey (NHANES) to assess the relationship among LPA, diet status and depression. Depression and LPA status was reported by the 9-item Patient Health Questionnaire (PHQ-9) and Physical Activity Questionnaire (PAQ), respectively. To assess dietary inflammatory preferences, dietary inflammatory index (DII) was applied based on a 24-h dietary recall interview. A total of 11,078 subjects was included in this study and weighted participants were 89,682,020. Weighted multivariable linear regression showed that DII was negatively associated with LPA after full adjustment, with β (95% CI): −0.487 (−0.647, −0.327). Weighted multivariable logistic regression showed that LPA was significantly associated with depressive symptoms after full adjustment, with odds ratios OR (95% CIs): 0.986 (0.977, 0.995). By DII stratification analysis, this phenomenon was also existed in groups with anti-inflammatory diet. Mediation effect analysis was further performed, which showed that DII significantly mediating the association between LPA and depression with proportion mediated as 3.94%. Our findings indicated the mediating role of DII in the association between LPA condition and incident depression. More well-designed studies are still needed to validate the causal relationship.

## Introduction

Depressive symptoms are common, heterogeneous conditions involving physical and psychological symptoms, and significantly produce health decrements (1, 2). Among non-fatal disease burden, depression will become the leading cause by 2030 according to WHO (3), whose prevalence is in excess of 300 million people (4), and it is estimated that major depressive disorder (MDD) has a lifetime prevalence (the proportion of one person who experienced the disorder at certain time during lifespan) of 16.6% (2). Combined with one of the symptoms being depressed mood, anxiety or absence of enjoyment, motivation, and interest, MDD is on the rise globally and now becoming the most concerned public health issues nowadays (5). In the United States, annual estimated highest expenditures to treat MDD could be up to $238.3 billion (6, 7), hence, depression not only brings mental disorders to human beings, but also may cause a large consumption of medical resources (8). Given the considerable influence of depression in general population, there is a pressing need to identify preventive or counter measures to deal with depression.

There is abundant evidence that the antidepressant effects could emerge with non-medical intervention represented by leisure-time physical activity (LPA) in teenagers' (9–11), middle-aged (12) and elder (13–15) groups. A recent meta-analysis, which included 49 unique prospective cohort studies and 266,939 participants, found that people with high levels of physical activity had lower odds of developing depression compared with low levels (adjusted OR = 0.83, 95% CI: 0.79 ~ 0.88; I^2^ = 0.00) (16). Simultaneously, other meta-analyses have demonstrated that benefits of physical activity for depression not only beyond risk reduction (17–19), but also can be regarded as a useful addition to pharmacotherapy and psychotherapy (16, 20), and also can reduce the risk of cardiovascular disease in people with depression (21). One cohort study which included 107,901 Korean adults have also indicated that when an appropriate level of physical activity is maintained, it can result in a positive effect by decreasing incident depression (22). In our previous research, we also concluded several biological and psychosocial mechanisms through which physical exercise exerted antidepressant effects, which further supplemented to explain the antidepressant effect of LPA (23, 24).

Nutrients have been established as a relevant factor in the development of depression. A large body of systematic review pointed out that pro-inflammatory diet was linked with increased risk of depression (25–27). Plentiful meta-analyses have also been put forward on the influence of diet on depression incidence, including but not limited to diet quality or diet pattern (27–30), and most of these phenomena might be explained by the effect of diverse dietary components. For instance, fiber, omega-3 fatty acids or saturated fats included in food can affect the incidence or strength of depression by inducing neuro-inflammation and stimulating changes in the oxidative stress, neurotransmitters and hypothalamic-pituitary-adrenal (HPA) axis (31, 32). The level of Interleukin 6 (IL-6), C-reactive protein (CRP) or other pro-inflammatory biomarkers can be enhanced by pro-inflammatory diets, and further raise the risk for the development of depression (32). However, people do not consume nutrients or food in isolation. In the past few years, scholars have increasingly emphasized the importance of diet as a whole (33, 34). Using the dietary index to quantify the inflammatory potential of the entire diet may increase the robustness and effectiveness of detecting the relationship between diseases (33). Recent study has demonstrated that dietary inflammatory index (DII) is inversely associated with depression (7). One meta-analysis showed that a 1-unit increment in DII was associated with an increased risk of 6% for depressive symptoms (35). Previous research also indicated that increased risk of depression was associated with the Western diet, characterized by consumption of high red and processed meat and refined carbohydrates, and decreased risk of depression with the Mediterranean diet, represented by high amount of fruit, vegetables, and whole grains (36, 37). In special group with chronic disease or comorbidity, there were higher prevalence of depression in groups with higher DII scores (38, 39).

Further studies have tentatively explored the joint effect of physical activity and diet on depression. Two recent randomized controlled trials indicated that a combined diet and exercise intervention can significantly led to positive alteration of depression, anxiety and quality of life in patients with cancer, no matter short or long term impact (40, 41). Another integrative review also showed that psychosocial well-being interacted with diet as well as with physical activity in women with Gestational Diabetes Mellitus (GDM) (42). Owing to depression also prime larger cytokine responses to stressors and pathogens that do not appear to habituate, negative health behaviors (e.g., poor diet, a sedentary lifestyle) may act as mediating pathways that lead to further, unrestrained inflammation and depression (43). However, there are still no investigations that take into account interactions between lifestyle factors (i.e., physical activity and dietary inflammatory preference) on depression by mediation analysis. A mediator is a third variable affecting the direction and/or strength of the relationship between a predictor and outcome variable (44). This mediator can thus act as a vulnerability or protective factor. Identifying mediator allows us to target the mediator in prevention and to identify at-risk populations.

Considering that evidence about the interactive effect of DII and physical activity on depression is limited and inconsistent, and it only concentrates on the small-scale and specific population. We aimed to use the National Health and Nutrition Examination Survey (NHANES) to assess associations between LPA and depressive symptoms under different levels of DII. Our hypothesis is that inadequate LPA and higher DII scores may promote the development of depression, while anti-inflammatory diet (lower DII scores) and increased physical activity are beneficial to alleviate depression.

## Methods

### Study population

As a nationally representative survey, the National Health and Nutrition Examination Survey (NHANES) is a cross-sectional survey organized by the National Center for Health Statistics at the U.S. Centers for Disease Control and Prevention. To represent for the civilian non-institutionalized U.S. population, a periodic 2-year cycle test with complex multistage probability sampling design was applied. Participants were asked to complete one or more of the following five sections: demographics, dietary, examination, laboratory, and questionnaire. The institutional review board approved the NHANES research protocol. The written and informed consent of all people participating in NHANES protocol followed the principles of the Declaration of Helsinki.

Data from six NHANES cycles 2007–2018 were enrolled in the present study. All data in this study were obtained from NHANES website, which was publicly available at https://www.cdc.gov/nchs/nhanes/ (accessed date: 10 May 2022). Initially, a total of 58,876 samples were included in. Participants without dietary information data to calculate DII (*n* = 74,04) were excluded. We further excluded participants with missing data on depressive symptoms (*n* = 21,107), and LPA (*n* = 16,331). After that, covariates with missing data were also filtered (*n* = 29,56). The final analytical sample thus was 11,078. The flow chart of the study sample was represented in Supplementary Figure S1.

### Exposure and outcome definitions

The exposure variable in this study was LPA and DII. LPA, which including sports, fitness and recreational activities, was assessed by the Physical Activity Questionnaire (PAQ) in NHANES (45). Participants self-reported their activity patterns through questions and was required to recall the type, frequency (exercise days per week) and duration (exercise times per day) of LPA they had undertaken during the past 7 day for a minimum of 10 min, including moderate and vigorous intensity bound up with recreational activities. For the definition of moderate/vigorous intensity LPA, it was described as recreational activities that required light/hard physical effort and cause minor/great increases in respiratory or heart rate. To evaluate the physiological intensity of LPA, the metabolic equivalents of task (MET) index was applied. For more details, MET was a unit to quantitatively assess the energy expenditure of one specific motion, where 1 MET refers to a consumption of 3.5 ml O_2_ kg^−1^/min (45). The suggested MET was 8.0 for vigorous LPA, while for moderate LPA was 4.0 (46). Considering that the change of 1 MET may not significantly reflect a cumulative effect of one-time exercise event, we used 100 MET as the unit to better describe the changing trends of RPA. Hence, in this study, RPA was assessed by 100 MET-min/week. We first multiplied the days spent performing certain activity by the mean duration by the suggested MET value and summed each activity values, and finally divided by 100 to obtain an estimate of total LPA.

The secondary exposure variable was the inflammatory potential of the diet, which was assessed using DII. The NHANES program and Nutrition Methodology Working Group obtained and validated dietary data at the mobile examination center. Subsequently, dietary components were documented and validated utilizing the 24-h dietary history interview to calculate DII scores based on the specific algorithm provided by N. Shivappa et al. (47). Briefly, the DII was evaluated using literature that assessed the association of dietary intake on six inflammatory biomarkers (i.e., IL-1β, IL-4, IL-6, IL-10, TNF-α, and CRP). Higher positive DII scores were regarded as more pro-inflammatory diet behavior, while lower negative DII ones indicated anti-inflammatory effects (47). In our study, 27 of the 45 food nutrients were available to calculate the DII score, including alcohol, β-carotene, caffeine, carbohydrates, cholesterol, energy, protein, total fat, fiber, folic acid, monounsaturated fatty acids, niacin, n-3 fatty acids, polyunsaturated fatty acids, riboflavin, saturated fat, thiamine, Fe, Mg, Se, Zn, vitamin A/C/D/E and vitamin B12/B6. Previous studies indicated that 27 or 28 of nutrients applied for the calculation would not affect the DII predictive capacity (48–50). Finally, the DII score was served as a continuous variable and then categorized into quantile 1, 2, and 3 (which represented for anti-inflammatory, some pro-inflammatory, and most pro-inflammatory diet) for further analysis.

As the outcome variable in this study, depressive symptom was assessed using the Patient Health Questionnaire-9 (PHQ-9) (51). PHQ-9 was a widely applied depression screening tool in non-psychiatric settings (52). Considering that PHQ-9 in NHANES was only applied for population over 18 years' age, participants included in our study were all adults. In the NHANES 2007-2018 cycle, the PHQ-9 was administered by the face-to-face Mobile Examination Centers (MEC) interview. Respondents reported their experience using a scale between “0” (not at all) and “3” (nearly every day) regarding each of the following nine questions, including anhedonia, depressed mood, sleep disturbance, fatigue, appetite changes, low self-esteem, concentration problems, psychomotor disturbances, and suicidal ideation. It was recommended that a total score > 10 was a screening cut-point for depression (53, 54), and this criterion has been proved to have a sensitivity and specificity of 88% to diagnose depression compared with mental health professional validation interviews (51).

### Covariate assessment

We incorporated age (year), sex, race/ethnicity, body mass index (BMI, kg/m^2^), education level, marital status, poverty status, smokers, alcohol drinkers, sleep duration, marital status, and chronic disease conditions (including hypertension, diabetes mellitus, and cardiovascular disease) as our covariates. Basic information on age, sex, race, education, and marital were obtained from the demographic interview. BMI was calculated based on weight and height measurement at MEC. Poverty status was evaluated by the poverty income ratio index. Data regarding smoking status and alcohol intake status were obtained from the questionnaires of smoking cigarette use and alcohol use. There was evidence that sleeping status might affect depression progress, hence we also included sleep duration as covariate. Sleep duration information was collected from self-reported questionnaire by participants. Chronic disease conditions were assessed by asking participants if they ever had been told they have high blood pressure, diabetes, and cardiovascular diseases (congestive heart failure, coronary heart disease, angina, heart attack or stroke).

### Statistical analysis

According to the CDC guidelines, sampling weights were employed and accounted for the complex multistage survey (sampling) design when statistical analysis were performed (55). For weighted characteristics description, continuous variables were presented as mean ± standard error and categorical variables were presented as percentages in this study. Weighted multivariable linear regression was used to investigate the association between LPA and DII. Weighted multivariable logistic regression was used to investigate the associations between LPA and depressive symptoms under different levels of DII. Since the percentage of missing data was small for any covariate, no imputation method was applied. To further explore the effect of covariates on this association, we employed Crude Model (unadjusted), Model 1 (age, sex, race/ethnicity were adjusted), and Model 2 (fully adjusted model). After assessing the direct effect of LPA on depression, we further investigated the indirect (mediating) effect of DII on this association. This analysis could assess how much percentage of the relationship between LPA and depression was mediated by the DII. The mediation package of R software was applied to conduct the regression-based mediation analysis. All statistical analysis tests were conducted using the software package R (http://www.R-project.org, The R Foundation). *P*-values < 0.05 were considered statistically significant.

## Results

### Population characteristics

Sociodemographic characteristics and related covariates of participants were presented in Table 1. After applying the selection and exclusion criteria, a total of 11,078 samples were finally included in, and the weighted population was 89,682,020. Roughly half of participants were male (51.38%). Over 70% of the participants were Non-Hispanic White and received college or above education. The median age was 44 years. The mean LPA was 1479 MET-minutes/week and mean DII score was 0.64 for our population. Participants with depressive symptoms were more likely to be female, BMI over 30 kg/m^2^, physical inactive and have more inflammatory diet (Table 1).

**Table 1:** Weighted characteristics of study populations in the NHANES (2007 – 2018) by depression.

**Variable**	**All participants**	**Non–depression**	**Depression**	***P–value***
**Age**				0.275
<44	29.53	29.40	31.95	
[44, 60]	39.03	38.95	40.67	
≥60	31.44	31.65	27.38	
**BMI(kg/m** ^**2**^ **)**				<0.001
<25	34.14	34.36	29.78	
[25, 30]	34.13	34.63	24.65	
≥30	31.73	31.00	45.58	
**Sex**				<0.001
Male	50.38	50.93	39.97	
Female	49.62	49.07	60.03	
**Race/ethnicity**				0.020
Non–hispanic White	72.00	72.27	67.00	
Non–hispanic Black	9.36	9.24	11.62	
Mexican American	6.37	6.35	6.69	
Other Race/ethnicity	12.26	12.14	14.70	
**Marital status**				<0.001
Never married	20.83	20.36	29.88	
Married/living with partner	63.79	64.77	45.04	
Widowed/ divorced	15.38	14.87	25.08	
**PIR**				<0.001
<1	10.31	9.58	24.13	
[1,3]	30.86	30.47	38.21	
≥3	58.84	59.95	37.66	
**Education**				<0.001
Below high school	2.07	2.02	3.00	
High school	24.04	23.38	36.7	
College or above	73.89	74.6	60.3	
**Smokers**				<0.001
Never smoker	60.36	61.09	46.58	
Former smoker	24.33	24.39	23.19	
Current smoker	15.31	14.52	30.23	
**Alcohol drinkers**				<0.001
Non-drinker	19.61	19.36	24.34	
Moderate alcohol use	61.53	62.07	51.3	
High alcohol use	18.86	18.57	24.36	
**Sleep duration (hour)**				<0.001
<7	60.70	61.62	43.20	
[7, 9]	29.03	28.28	43.28	
≥9	10.27	10.10	13.52	
LPA (100MET–minutes/week, continuous)	14.79 ± 0.19	14.92 ± 0.20	12.30 ± 0.58	<0.001
DII (continuous)	0.64 ± 0.04	0.61 ± 0.04	1.29 ± 0.12	<0.001
**DII (category)**				<0.001
Anti–inflammatory diet	36.91	37.37	28.04	
Some pro–inflammatory diet	33.04	33.3	28.06	
Most pro–inflammatory diet	30.05	29.33	43.90	
**Hypertension**				0.014
No	69.24	69.61	62.13	
Yes	30.76	30.39	37.87	
**DM**				0.015
No	89.03	89.25	84.89	
Yes	10.97	10.75	15.11	
**CVD**				0.011
No	94.61	94.79	91.07	
Yes	5.39	5.21	8.93	

Mean ± SE for continuous variables: P-value was calculated by weighted linear regression model. % for categorical variables: P-value was calculated by weighted chi–square test. NHANES, National Health and Nutrition Examination Survey; BMI, body mass index; PIR, poverty income ratio; LPA, leisure–time physical activity; DII, dietary inflammatory index; DM, diabetes mellitus; CVD, cardiovascular diseases.

### LPA status by DII quartiles

**Figure 1:**
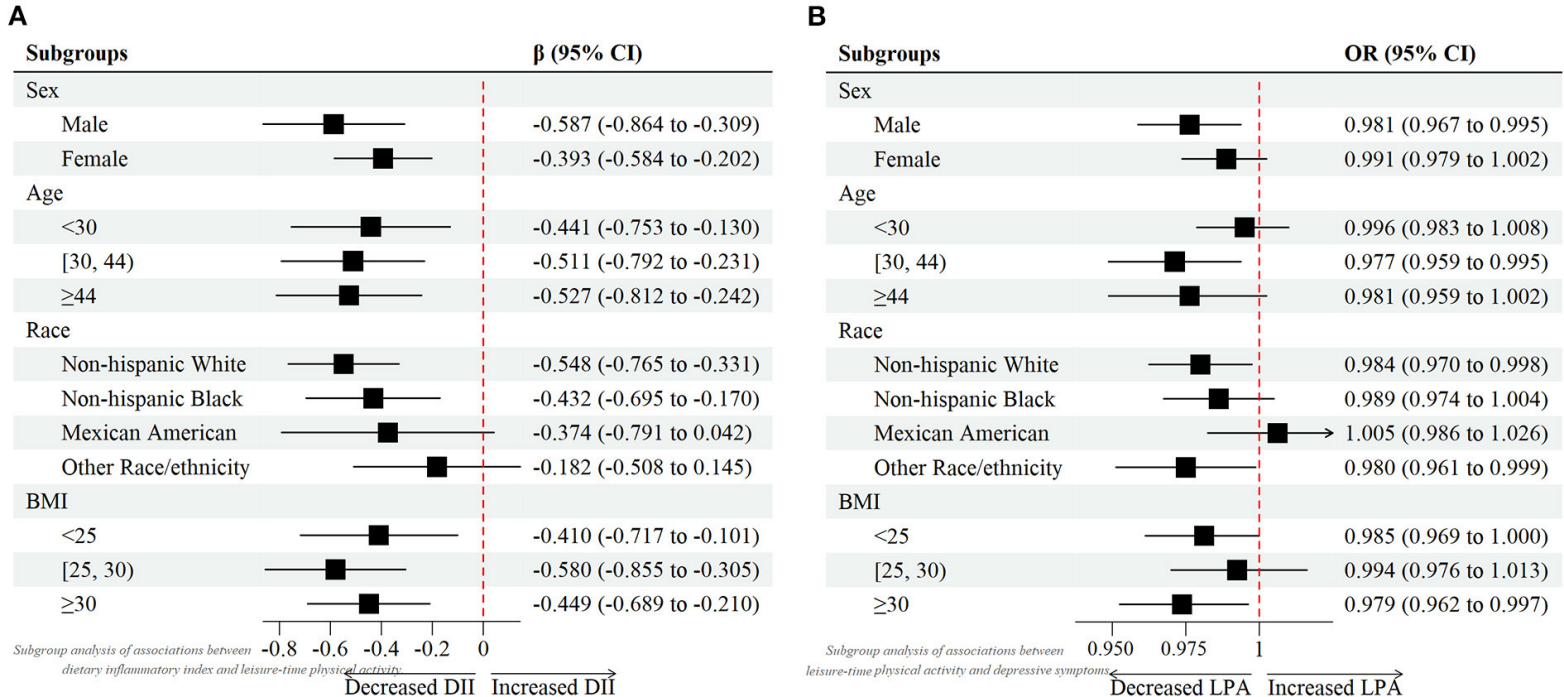
Forest plots of **(A)** associations between dietary inflammatory index and leisure-time physical activity. **(B)** associations between leisure-time physical activity and depressive symptoms.

Weighted multivariable linear regression model was employed to explore the association between DII and LPA status (Table 2). When DII was assessed as continuous variable, results revealed that higher DII was associated with less LPA amount (Crude Model, β = −0.686, 95% CI: −0.831, −0.542, *p* < 0.001; Model 1, β = −0.600, 95% CI: −0.758, −0.442, *p* < 0.001; Model 2, β = −0.487, 95% CI: −0.647, −0.327, *p* < 0.001). When DII was assessed as category variable, this association persisted as well. In the fully adjusted Model 2, taken quantile 1 of DII (anti–inflammatory diet) as the reference, some and more pro–inflammatory diet was negatively associated with LPA level (Q2 vs. Q1 β: −1.840; 95% CI: −2.607, −1.072; *p* < 0.001; and Q3 vs. Q1 β: −1.958; 95% CI: −2.742, −1.173; *p* < 0.001, respectively). In addition, through subgroup analysis, forest plot (Figure 1A) demonstrated that the negative association between DII score and LPA was consistent across four groups. This indicated that participants with more inflammatory diet tended to be involved in less exercise or recreational physical activities in their life.

**Table 2:** Associations between leisure–time physical activity and dietary inflammatory index.

**LPA (100MET–minutes/week)**	**Crude model** ^a^		**Model 1** ^b^		**Model 2** ^c^	
	**β (95% CI)**	***P–value***	**β (95% CI)**	***P–value***	**β (95% CI)**	***P–value***
DII as continuous variable	−0.686(−0.831,−0.542)	<0.001	−0.600(−0.758,−0.442)	<0.001	−0.487(−0.647,−0.327)	<0.001
DII as category variable						
Anti–inflammatory diet	Reference		Reference		Reference	
Some pro–inflammatory diet	−2.333(−3.097,−1.570)	<0.001	−2.172(−2.945,−1.399)	<0.001	−1.840(−2.607,−1.072)	<0.001
Most pro–inflammatory diet	−2.913(−3.625,−2.202)	<0.001	−2.516(−3.290,−1.743)	<0.001	−1.958(−2.742,−1.173)	<0.001

a Crude model, no covariates were adjusted.

b Model 1, age, sex, race/ethnicity were adjusted.

c Model 2, age, sex, race/ethnicity, body mass index, education marital status, poverty status, smokers, alcohol drinkers, sleep duration and chronic disease conditions were adjusted. LPA, leisure–time physical activity; DII, dietary inflammatory index; CI, confidence interval.

### Depression by LPA status under different DII quartiles

Weighted multivariable logistic regression model was used to investigate the relationship between LPA status and depression under different DII levels (Table 3). First, we assessed the relationship between LPA and depression in total samples. Results showed a consistent negative trend between LPA and depression in three different models (Crude Model, OR = 0.983, 95% CI: 0.974, 0.992, *p* < 0.001; Model 1, OR = 0.983, 95% CI: 0.974, 0.993, *p* < 0.001; Model 2, OR = 0.986, 95% CI: 0.977 0.995, *p* < 0.001). When Stratified by DII, similar results were found in the anti–inflammatory diet group in the fully adjusted model (OR = 0.976, 95% CI: 0.962, 0.990, *p* < 0.001). However, in the some and more pro–inflammatory diet group, although a similar negative association was found, the statistics seemed to be not significant (Model 2, OR = 0.998, 95% CI: 0.981, 1.014, *p* = 0.795; OR = 0.986, 95% CI: 0.971, 1.001, *p* = 0.065, respectively). This suggested that more dietary inflammatory tendency might attenuated the effects of LPA on the odds of probable depression. Additionally, considering on the age, gender, race influence, as well as BMI on depression prevalence, subgroup analyses (Figure 1B) were also performed to further examine the relationship between LPA and depression in different groups.

**Table 3:** Associations between leisure–time physical activity and depressive symptoms under different levels of DII.

**LPA (100MET–minutes/week)**	**Crude model** ^a^		**Model 1** ^b^		**Model 2** ^c^	
	**OR (95% CI)**	***P–value***	**OR (95% CI)**	***P–value***	**OR (95% CI)**	***P–value***
Total samples	0.983(0.974,0.992)	<0.001	0.983(0.974,0.993)	<0.001	0.986(0.977,0.995)	0.004
Stratified by DII						
Anti–inflammatory diet	0.974(0.959,0.990)	0.001	0.973(0.959,0.988)	<0.001	0.976(0.962,0.990)	0.002
Some pro–inflammatory diet	0.993(0.978,1.008)	0.341	0.995(0.979,1.011)	0.514	0.998(0.981,1.014)	0.795
Most pro–inflammatory diet	0.986(0.972,1.001)	0.075	0.985(0.970,1.001)	0.064	0.986(0.971,1.001)	0.065

a Crude model, no covariates were adjusted.

b Model 1, age, sex, race/ethnicity were adjusted.

c Model 2, age, sex, race/ethnicity, body mass index, education marital status, poverty status, smokers, alcohol drinkers, sleep duration and chronic disease conditions were adjusted. LPA, leisure–time physical activity; DII, dietary inflammatory index; OR, odds ratio; CI, confidence interval.

### Mediation analysis

**Figure 2:**
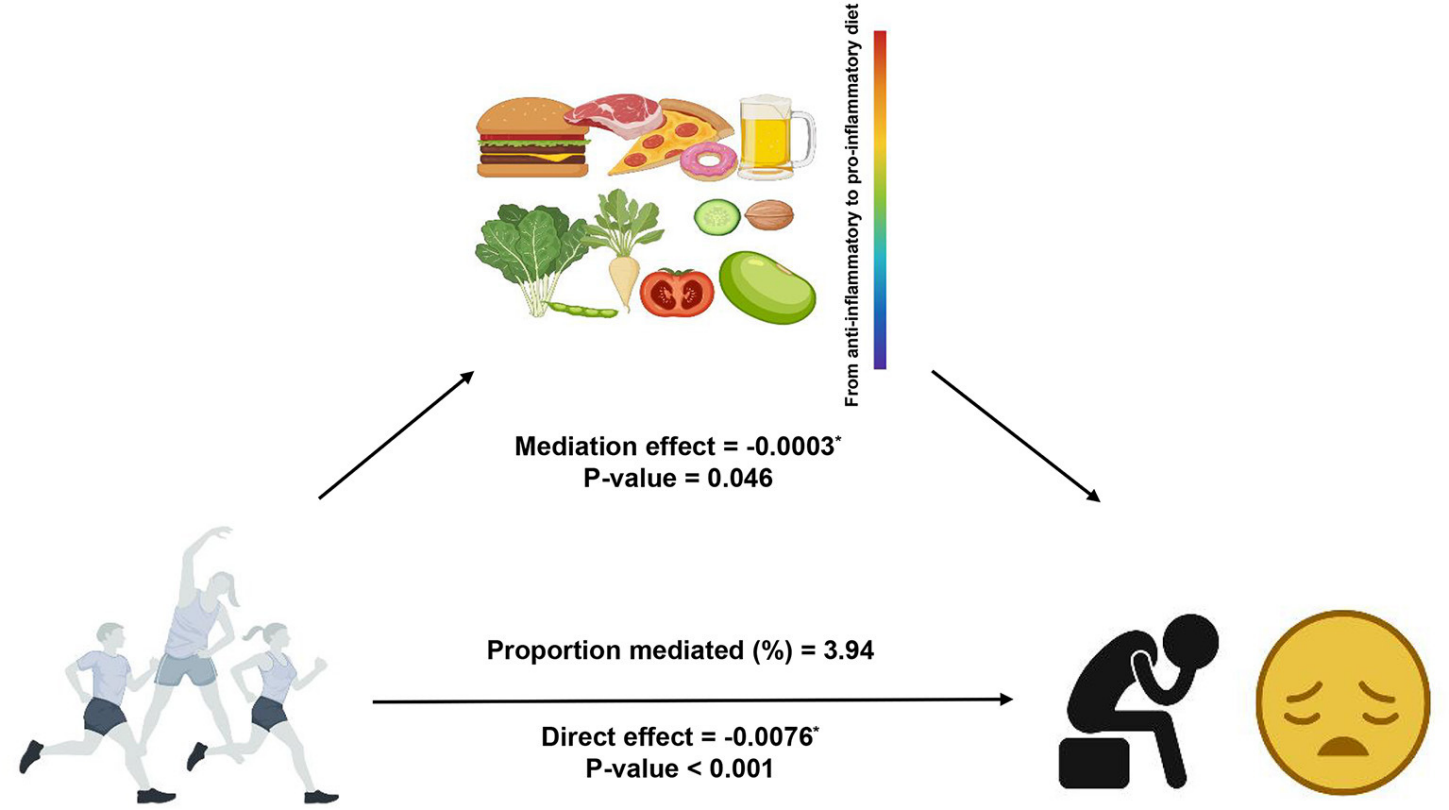
Path diagram of the mediation analysis model.

The result of the mediation analysis was shown in Figure 2. The outcome variable was depression, the exposure variable was LPA status and the mediator variable was DII. We used Model 2 (fully adjusted model) to conduct the mediation analysis. The total effect on the total depression score was −0.0079 (*p* < 0.001), of which the direct effect of LPA was −0.0076 (*p* < 0.001), and −0.0003 was explained by the indirect effect of DII (*p* = 0.046). Significant indirect effect of DII was observed and 3.94% of total change was attributed to the mediation effect of DII.

## Discussion

On the basis of the analysis of 11,078 samples in the NHANES 2007–2018 cycle, this current study for the first time reported the relationship between LPA and depressive symptoms under different levels of DII and verified the mediation effect of DII in this association. We firstly found a negative relationship between the baseline LPA status and DII level which indicated that groups with higher DII scores tended to participate in less physical activities. Subsequently, we detected a negative association between LPA and depressive symptoms while this finding statistically held only in the anti–inflammatory diet group (corresponding to a lower DII scores). Furthermore, our mediation analysis showed that the association between LPA and depression was partly mediated by DII.

Our findings on the relationships between LPA, DII and depressive symptoms supported and extended those from previous publications. As a behavioral phenotype, being physically inactive or keeping more sedentary time has been proved to be connected with low to moderate grade systemic inflammation (56, 57). One cross–sectional study carried among European teenagers assessed that a healthy diet might attenuate the positive sedentary time and inflammation relation (58). Importantly, the prevalence of these physical inactive behavior increased not only through adolescence, and once installed in this phase, there was a strong tendency it remained into adulthood (59). In adults across a wide age spectrum from 20–85 years, one previous study further reported the association between physical activity and dietary inflammatory index on mortality risk in U.S. adults (60). In this research, the author suggested that the combination of consuming a more anti–inflammatory diet and having adequate levels of physical activity can reduce the risk of mortality. Compared with the former research, our research was an improvement which further detected that groups with higher DII scores tended to be less physically active. Our findings, if demonstrated in other large–scale prospective studies, may provide information that with the increment of DII, people not only did not tend to do more LPA to rescue, but also become more physically inactive.

When it comes to depression, prolonged physical inactivity were important risk factors. Behavioral intervention such as increasing moderate–to–vigorous exercise has been detected to exhibit approximate antidepressant effects (17, 61), which was consistent with our findings that doing more LPA was correlated with lower emergence of depression. From the mechanism, 'myokines' (cytokines produced or released by muscle) played an important role, evidence showed that muscle–induced peripheral factors can induce direct crosstalk between muscle and brain function, including the effects on depressive symptoms (62–64). From the dose–effect perspective, one meta–analytic evidence from prospective cohort studies proved that doing moderate–to–vigorous physical activity around 150 min per week would have a protective effect against prevalence of depression (16). However, these evidence might be inconsistent in different dietary preference groups. One research showed that obesity is a risk factor connected with depression and could to some tent be prevented by accomplishing more LPA and low energy–dense diet (65). In accordance with the previous finding, our study also detected that in groups with anti–inflammatory diet, LPA can significantly reduce the prevalence of depression. It seemed that increasing LPA, and keeping anti–inflammatory dietary habits can subsequently prevent obesity, which might be helpful in struggling with psychosocial stress including depression.

With respect to the joint effect of LPA and dietary inflammation on depression, little is known about the distinct biological mechanisms, while inflammatory biomarkers seemed to play an important role. A growing number of publications have reported that exercise can induce considerable physiological adaptions in the immune system, cytokines (i.e., IL−6, IL−8, IL−10, and TNF–α), and stress hormones (66–68), which was beneficial to achieve similar effects with antidepressants. On the other hand, pro–inflammatory dietary behavior might involve in the modulation of pathways including oxidative stress, epigenetics and mitochondrial dysfunction (69). Recent evidence also put into the fact that higher dietary inflammation led to the leaky gut and bacterial translocation (70, 71), which may eventually promote depressive symptoms. Hence it is of great significance to examine the risk–benefit relationship between LPA and DII on depression. Our results suggested that diet inflammation, indicated by DII score, could mediated the negative association between LPA and depression, which provide convincing evidence on the hypothesized complex relationships. One latest evidence published in *Nature* detected that exercise–inducible metabolite can control food intake and influence systemic energy balance, which might partially support the mediation effects of dietary from the mechanism perspective (72). The combination of performing adequate level of physical activity and consuming a more anti–inflammatory diet might have positive effects on depressive symptoms, and there is necessity of clarifying the in–depth mechanisms underlying these relationships.

Yet unfortunately, no consensus has been reached that whether LPA was effective in preventing depression in individuals with higher dietary inflammation. Our statistical analysis showed that although there was a negative association between LPA and depression in the higher DII scores groups, this relationship was not significant. Given the abundant evidence supporting correlations between dietary inflammation (73) and physical exercise (74) with inflammatory biomarkers as well as the evidence for the role of inflammation in the development of depression, it led us to propose that the dose–effect relationship served as an important factor that affect the association between these lifestyle behaviors (i.e., diet and exercise patterns) with depression conditions. However, such provocative hypothesis was not entirely validated and future researches could assess these relationships in a dosage–dependent manner.

There were multiple strengths in this current study. Notably, the present study not only extended existing evidence but further highlighted the association between LPA and depression under different levels of DII, thereby further strengthening the possible application of LPA and DII in large population to prevent depression. Another key strength of our study was that it included a large sample size representing the general US population. Further, whereas previous studies have looked at the relationship of physical activity or dietary condition and depression, this current study took an innovative approach which to our knowledge was the first to examine the mediating effect of DII on the association between LPA and depression. This is important because it directed further studies and dietary interventions to consider relationship between physical exercise and depressive symptoms.

Our study had several limitations. Due to nature of this cross–sectional study design, we were temporally not able to deduce the causal inference. Secondly, depression data and LPA assessment in our study were based on self–reported questionnaire. However, PHQ9 and PAQ were regarded as “gold standard” criteria for large population–based studies, which has been applied in several previous publications (75, 76). In addition, DII score calculated by 24–h dietary recall may not only cause imprecise estimates due to inability in measuring day–to–day variability in dietary intake, but also characterize population or group intake rather than a measure of individual intake (77). Nutritional supplements should be considered as another impact factor that influence DII status. Furthermore, only BMI was reported as an indication for body composition in our analysis, and bioimpedance was considered as a more accurate and objective option. Recently, the comorbidity of obesity and depression received new attention (78–80), and the in–depth mechanisms regarding to inflammation and the gut microbiota are worthy of further exploration. To fully explore the comprehensive links between LPA, DII and depression, a longitudinal study with accurate and complete assessment of exercise and dietary behavior was needed.

## Conclusion

In summary, our findings Indicated that (i) groups with higher DII scores tended to participate in less physical activities; (ii) performing more LPA was associated with a lower risk of depression in the anti–inflammatory diet groups; and (iii) DII was an important factor to mediate the association between LPA and depression. Our results could provide guidance for the development of prevention strategies with increased emphasis on the overall effect of physical activity status and diets inflammation for the prevalence of depression. Ultimately, findings of our study aligned with the idea that a comprehensive lifestyle management including conducting regular exercise and consuming a more anti–inflammatory diet would be beneficial to effectively reduce depression symptoms in the general population. These findings warranted further longitudinal and experimental research.

## Data availability statement

Publicly available datasets were analyzed in this study. This data can be found here: https://www.cdc.gov/nchs/nhanes/ (accessed date: 10 May 2022).

## Ethics statement

The studies involving human participants were reviewed and approved by National Center for Health Statistics (NCHS) and the NCHS Institutional Review Board (IRB). The patients/participants provided their written informed consent to participate in this study.

## Author contributions

Conceptualization and formal analysis: YY, YC, and JY. Methodology, software, and writing—original draft preparation: YY and YC. Investigation: ZZ, KZ, JZ, and SJ. Writing—review and editing: YY, YC, JY, ZZ, and KZ. Supervision: JZ and SJ. All authors have read and agreed to the published version of the manuscript.

## Conflict of interest

The authors declare that the research was conducted in the absence of any commercial or financial relationships that could be construed as a potential conflict of interest.

## Publisher's note

All claims expressed in this article are solely those of the authors and do not necessarily represent those of their affiliated organizations, or those of the publisher, the editors and the reviewers. Any product that may be evaluated in this article, or claim that may be made by its manufacturer, is not guaranteed or endorsed by the publisher.
