# Supplementary figures and images for: Relationship between leisure-time physical activity and depressive symptoms under different levels of dietary inflammatory index
Source: Front Nutr. 2022 Sep 7;9:983511. doi: 10.3389/fnut.2022.983511 (PMC9490084; doi:10.3389/fnut.2022.983511)

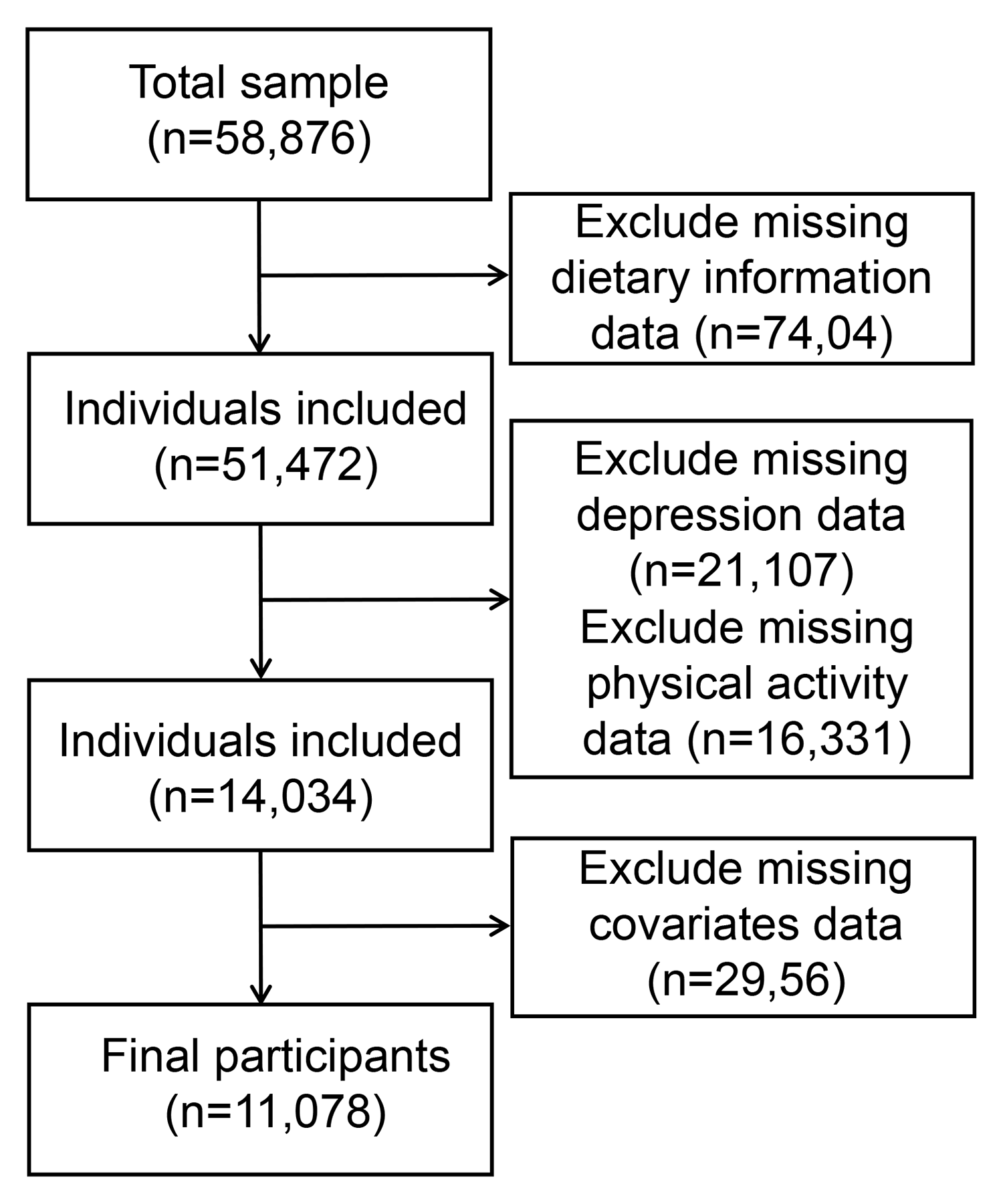

Supplement: Supplementary file 1 [file Image_1.TIF]
